# Comparing the effects of premarital booklet‐ and video‐based educations on the reproductive health literacy of engaged couples

**DOI:** 10.1002/hsr2.70116

**Published:** 2024-10-06

**Authors:** Sanaz Bahrami‐Samani, Farideh Mohsenzadeh–Ledari, Shabnam Omidvar, Soraya Khafri, Hoda Mohsenian

**Affiliations:** ^1^ Student Research Committee Babol University of Medical Sciences Babol Iran; ^2^ Social Determinants of Health Research Center, Health Research Institute Babol University of Medical Sciences Babol Iran; ^3^ Department of Biostatistics and Epidemiology, School of Public Health Babol University of Medical Sciences Babol Iran; ^4^ Population, Family and School Health Department, Health Deputy Babol University of Medical Sciences Babol Iran

**Keywords:** couples, Health literacy, Marriage, premarital education, Reproductive health

## Abstract

**Objectives:**

The aim of this study was to compare the effects of premarital booklet‐based education (BBE) and video‐based education (VBE) on engaged couples’ RHL.

**Methods:**

This quasi‐experimental study was conducted in 2021–2022. The study population consisted of engaged couples who referred to Amirkola Premarital Counseling Center, Babol, Iran, to receive premarital counseling. One hundred and ten couples were purposefully recruited and randomly allocated to a 55‐couple BBE group and a 55‐couple VBE group. Participants in the BBE group received RH‐related education through an educational booklet, while participants in the VBE group received RH‐related education through 4 weekly RH‐related educational videos shared through WhatsApp. The Sexual and Reproductive Health Literacy questionnaire was used to assess RHL at three measurement time points, i.e., before, 4 weeks after, and 2 months after the intervention onset. The data were analyzed using the SPSS software (v. 23.0).

**Results:**

The mean score of RHL significantly increased in both BBE and VBE groups (*p* < 0.001). The time‐group interaction was also significant for the mean scores of the access to information, evaluation of information, and decision‐making and application dimensions (*p* < 0.05) and insignificant for the reading and understanding dimension of RHL (*p* = 0.56).

**Conclusion:**

Both premarital BBE and VBE are effective in significantly improving RHL among engaged couples, while the effects of VBE on some RHL dimensions are significantly more than BBE.

## 1 INTRODUCTION

Reproductive health (RH) is one of the main aspects of health and one of the main determinants of the health of generations. It is a state of complete physical, mental, and social well‐being and is not the mere absence of illness or infirmity respecting the functions and process of the reproductive system. RH depends on a positive and respectful approach to sexuality and sexual relationships, enjoyment of safe and pleasurable sexual relationships, and respectful fulfillment of the sexual rights of all people, without any coercion, discrimination, and violence.^1^

RH literacy (RHL) is a key determinant of RH. It refers to a set of personal knowledge, attitudes, beliefs, motivations, and abilities to acquire, understand, evaluate, and use information about reproductive and sexual health in daily life to negotiate, judge, and decide about sexual healthcare, health promotion, relationships, and well‐being.^2^ According to the World Health Organization, RHL is a spectrum of literacy respecting reproductive and sexual health that includes different dimensions such as sexual development, puberty, reproduction, contraception, unwanted pregnancy, sexually transmitted diseases, and development of sexual relationship management skills.^1^, ^3^ Health literacy (HL) refers to the ability to access available healthcare services, understand and use health‐related information, interpret health‐related issues, and make appropriate health‐related decisions.^4^ It is a cognitive and social skill, its dimensions are understanding information, evaluating it, accessing it, and decision making, and consisted of a set of skills such as reading, writing, listening, and analyzing health‐related information and using these skills in health‐related situations.^5^ The World Health Organization considers RHL as one of the main determinants of health^6^ and recent studies show RHL improvement as one of the most important strategies to attain the goals of RH.^7^, ^8^, ^9^

Adequate RHL improves the ability to evaluate and understand sexual health risks, postpones the first sexual experience, contributes to engagement in safe sexual activity, prevents sexually transmitted diseases, improves understanding about responsibilities in sexual relationships, provides appropriate opportunities to accurately perform gender‐specific roles, and improves personal sexual health and familial and social health.^9^ Evidence shows that most adverse health‐related outcomes are due to inadequate HL and highlights the importance of HL improvement.^10^ People with limited HL are unable to effectively communicate with healthcare providers and hence, may not be able to make sound health‐related decisions.^11^ Furthermore, they are less likely to accurately understand written and verbal information provided by healthcare providers and have poor adherence to their recommendations.^12^ Moreover, limited HL is associated with problems in using protective and screening health‐related services, low disease management knowledge, high healthcare costs, high number of medical visits, high prevalence of chronic diseases, and poor health status.^13^

Education is one of the influential factors on HL and hence, is considered as a key component of HL improvement programs.^14^ Education can improve clients’ understanding ofavailable healthcare services, improve their knowledge about the contributing factors of health and illness, enhance their health responsibility, and enable them to make informed decisions about using healthcare services.^15^ According to the World Health Organization, education is associated with permanent change in knowledge, attitude, performance, and lifestyle.^14^ Previous studies also confirmed that education has significant positive effects on health^16^ and improves health‐related knowledge and HL.^17^, ^18^ Education about sexual and reproductive issues potentially improves RH and RH‐related behaviors and reduces familial and marital problems.^19^ Improvement of RH and RH‐behaviors can in turn improve marital relationships.^20^ Consequently, premarital education is considered as a key component of premarital counseling to improve couples’ knowledge aboutRH.^19^

There are different methods for health education provision. One of these methods is self‐learning through written materials. In this method, learners can receive education and improve their knowledge based on their own time schedule,^21^ interests, and needs^22^ and without any need for in‐person attendance at a determined place.^21^ This method improves learners’ self‐confidence and personal learning abilities.^23^ Books, booklets, and magazines are the most commonly used materials for self‐learning through written materials. Booklets are among the most primary sources of information used in self‐learning and include short pieces of conceptual information about a certain topic and are developed for specific target groups. However, some studies showed that booklet‐based education (BBE) is associated with limited learner‐instructor interaction, is not appropriate for learners with low educational level, and hence, cannot be considered as an independent method for education.^21^, ^24^

Virtual education is another method for client education in healthcare settings. This modern method has overcome some shortcomings of traditional methods and facilitated easy and flexible access to educational materials anywhere, even in remote areas.^25^ It has significantly reduced the costs and the time needed for education, facilitated the interchange of information and skills, and provided the opportunity to provide any educational material to any person anytime and anywhere.^26^, ^27^ One of the strategies to provide virtual education is video‐based education (VBE). In VBE, educational videos are used to facilitate learning and deepen learners’ understanding ofeducational materials.^28^ This method has its own shortcomings such as the large amount of time needed to make educational videos, the difficulty of updating them, and the limitation of face‐to‐face instructor‐learner interactions.^28^, ^29^

Previous studies reported different and even contradictory results respecting the effects of education on RHL. For example, a study on female school students in Thailand showed that education through cell phone messages had significant positive effects on sexual and reproductive HL.^30^ However, two studies showed that premarital educational programs had no significant effects on RH‐related knowledge and attitude.^31^, ^32^ Moreover, studies in Iran showed that despite the availability of appropriate and up‐to‐date educational materials for premarital education, these materials are not effectively provided to couples.^31^, ^32^ Besides, to the best our knowledge, there is no comparative study into the effects of virtual premarital education on RHL. Therefore, the present study was carried out to produce more evidence in this area. The aim of this study was to compare the effects of premarital BBE and VBE on engaged couples’ RHL.The occurrence of the COVID‐19 and the subsequently decrease in the possibility of attending and receiving training in person was one of the reasons for this investigation and comparing the virtual method with the self‐study method.

## 2 METHODS

This cross‐sectional interventional study was conducted from August 2021 to February 2022.

### 2.1 Participants and setting

**Figure 1:**
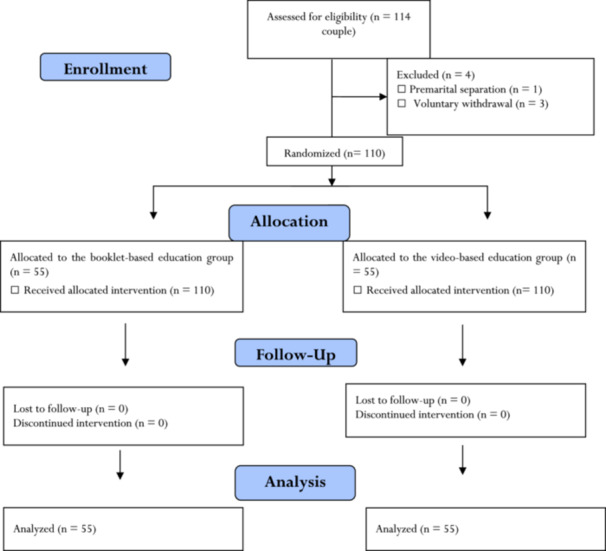
The flow diagram of the study.

Study setting was Amirkola Premarital Counseling Center affiliated to Babol University of Medical Sciences, Babol, Iran, and the study population consisted of engaged couples who referred to this center to receive premarital counseling. Sampling was purposefully performed based on the following selection criteria: Iranian nationality, basic literacy skills, ability to use online social networks, no previous history of marriage, consent for participation, and no affliction by any learning disability or mental disorder. Exclusion criteria were incomplete use of educational materials and voluntary withdrawal from the study. Participants were randomly allocated to a BBE group and a VBE group through block randomization with a block size of 4. A list of random numbers was created for randomization using the Microsoft Excel software (2016) and a numerical code was allocated to either of the study groups. Only the first author of the study was aware of the group codes. Figure 1 shows the study flowchart.

Sample size was calculated based on the results of a previous study and with an effect size of 0.57,^33^ a power of 0.80, and a confidence level of 0.95. Accordingly, 110 couples (55 couples per group) were determined to be necessary.

### 2.2 Instruments

Data collection instruments were a demographic questionnaire and the Sexual and Reproductive Health Literacy questionnaire. The demographic questionnaire contained items on age, educational level, place of birth and residence, employment status, and income. The Sexual and Reproductive Health Literacy questionnaire, introduced by the World Health Organization,^34^ is a 37‐item questionnaire with four dimensions, namely reading and understanding (nine items), access to information (twelve items), evaluation of information (six items), and decision making and application (ten items). Items are scored on a five‐point 1–5 Likert scale and hence, the possible total score of the questionnaire is 37–185. Dabiri et al., confirmed the acceptable validity and reliability of this questionnaire in Iran with a content validity ratio of 0.92, a content validity index of 0.98, and a test‐retest intraclass correlation coefficient of 0.86.^33^ In the present study, participants in both groups completed this questionnaire at three time points, namely before, four weeks after, and two months after the intervention onset (T1–T3).

### 2.3 Intervention

Educational materials for both groups were the same and were developed based on the sexual and RH educational booklets provided by the Ministry of Health of Iran. Participants in the BBE group were provided with a sexual and RH educational booklet for self‐study. They were also added to a WhatsApp group for sexual and reproductive HL assessment at T2 and T3. Participants in the VBE group received sexual and RH‐related education through educational videos provided to them through WhatsApp. Initially, a WhatsApp group for female participants and a WhatsApp group for male participants were created and then, one educational video was uploaded for them every 1 week for four consecutive weeks (four videos in total). There were discussion processes between the researcher and the intervention group on a regular basis (to monitor and ensure whether they consistently follow the intervention program provided. They were also invited to complete the sexual and reproductive HL assessment questionnaire online at T2 and T3. The content of the videos was as follows:

### 2.4 Data analysis

The main outcome of the study was RHL and the data were analyzed using the SPSS software (v. 23.0). Between‐group comparisons were performed using the Chi‐square and the independent‐sample *t* tests, while within‐group comparisons were performed through the repeated measures analysis of variance. Pairwise comparisons among the three measurement time points were also performed using the Bonferroni's method. The level of significance was set at less than 0.05. Table 1.

**Table 1:** Contents of the sessions.

Sessions	Content	Target
**First**	1. The importance of marriage and maintaining self‐respect. 2. Promotion and development of positive characteristics such as optimism, mutual kindness, and the ability to communicate well and maintain the relationships. 3. Considering times for concerted activities, such as: watching TV, Talking about daily experiences, sports and cooking etc. 4. Maintaining mutual respect of each other's families, even if there are differences in the opinions. 5. Identifying the value and abilities of the spouse and respect to this value.	Key points about having a stable and happy family
**Second**	1. Getting to know the shape and structure and types of hymen. 2. Evaluation of individual information regarding sexual appearance and performance and correct sexual beliefs. 3. Preparation for sexual relationship. 4. Explanations about the sexual response cycle, including: desire, arousal, intercourse, orgasm and withdrawal. 5. Explanations about the sensitive areas of women and men. Explanations about contraceptive methods such as: combined contraceptive pills, condoms to prevent unintended pregnancy	Overview of the genital system, sexual function, and contraception
**Third**	1. Explanations about menstrual cycle. 2. Explanation about menstrual hygiene. 3. Brief explanations about fertilization and embryo development. 4. Key points about having a healthy and safe fertility.	Reproductive health and care
**Fourth**	1. The importance of age in fertility and raising a healthy fetus. 2. Complication of pregnancy. 3. Dangerous complications of abortion. 4. Preparation for childbirth. 5. Advantages of natural childbirth and disadvantages of unnecessary cesarean section	Critical pregnancy care and childbearing

Demographic variables were included in the analysis as confounding variables and were measured using univariate tests between two groups and there was no significant difference.

### 2.5 Ethical considerations

The Ethics Committee of Babol University of Medical Sciences, Babol, Iran, approved this study. Clear explanations about the study's aim and methods were provided to all participants and they were asked to provide written informed consent for participation.

## 3 FINDINGS

Initially, 114 couples (i.e., 228 individuals) were recruited to the study. Four couples were excluded due to premarital separation (*n* = 1) and voluntary withdrawal (*n* = 3). Consequently, 110 couples in two 55‐couple groups completed the study.

The mean of participants’ age was 23.56 ± 4.7 years in the BBE group and 23.68 ± 4.4 years in the VBE group. Most participants in these groups had been born in urban areas (70.9% vs. 82.7%) and almost half of them lived in urban areas (52.7% vs. 49.1%). Most participants in both groups considered their income sufficient (72.7% vs. 62.7%). Most participants of the groups were Educated (University level) (72.8% vs.62.8%) and 50.9% of the women in VBE group and 61.8% in BBE group were housewife. There were no significant differences between the groups respecting participants’ demographic characteristics (*p* > 0.05; Table 2).

**Table 2:** Between‐group comparisons respecting participants’ demographic characteristics.

Group				
Characteristics		Video‐based education	Booklet‐based education	*P* value
	Age (Years) [Mean ± SD (Range)]	23.68 ± 4.4 (15–34)	23.56 ± 4.7 (13–36)	0.84*
Educational level [*N* (%)]	High school	5 (4.5)	7 (6.4)	0.327**
	Diploma	25 (22.7)	34 (30.9)	
	Bachelor's	69 (62.8)	63 (57.3)	
	Master's and higher	11 (10)	6 (5.5)	
Place of birth [*N* (%)]	Urban areas	91 (82.7)	78 (70.9)	0.055**
	Rural areas	19 (17.3)	32 (29.1)	
Place of residence [*N* (%)]	Urban areas	54 (49.1)	58 (52.7)	0.68**
	Rural areas	56 (50.1)	52 (47.3)	
Occupation (Female participants) [*N* (%)]	Unemployed	28 (50.9)	34 (61.8)	0.167**
	Indoor	9 (16.4)	3 (5.5)	
	Outdoor job	18 (32.7)	18 (32.7)	
Occupation (Male participants) [*N* (%)]	Unemployed	6 (10.9)	7 (12.8)	0.16**
	Employed	49 (89.1)	48 (87.2)	
Income status [*N* (%)]	Sufficient	69 (62.7)	80 (72.7)	0.16**
	Quite sufficient	35 (31.8)	28 (25.2)	
	Insufficient	6 (5.5)	2 (8.1)	

* The results of the independent‐sample *t* test.

** The results of the Chi‐square test.

Between‐group comparisons revealed that the total mean score of RHL in the VBE group was significantly more than the BBE group at all three measurement time points (*p* < 0.05). Moreover, within‐group comparisons indicated significant increase in the total mean score of RHL in both groups across the three measurement time points (*p* < 0.001). However, the amount of increase in the VBE group was significantly more than the BBE group (*p* < 0.001). The interaction of time and group was also significant (*p* = 0.002).

Between‐group comparisons indicated that the mean scores of the reading and understanding and the evaluation of information dimensions of RHL in the VBE group were significantly more than the BBE group at all measurement time points (*p* < 0.05). Moreover, while there were no significant between‐group differences respecting the pretest mean scores of the access to information and the decision making and application dimensions of RHL (*p* > 0.05), the mean scores of these two dimensions in the VBE group were significantly more than the BBE group at T2 and T3 (*p* < 0.05).

Within‐group comparisons also indicated that the mean scores of all four dimensions of RHL significantly increased in both groups across the three measurement time points (*p* < 0.05) and the amount of increase in the VBE group was significantly more than the BBE group (*p* < 0.05). Moreover, the time‐group interaction was significant for the mean scores of the access to information, evaluation of information, and decision‐making and application dimensions (*p* < 0.05) and insignificant for the reading and understanding dimension (*p* = 0.56) (Table 3).

**Table 3:** Within‐ and between‐group comparisons respecting the mean scores of reproductive health literacy and its dimensions.

Outcomes	Time Group	Before	Four weeks after	Two months after	*P* value**		
					Time	Group	Time‐Group
Total	VBE	144.28 ± 24.95^A^	158.19 ± 16.21^B^	168.78 ± 11.50^C^	<0.001	<0.001	*F* = 6.83 *p* = 0.002
	BBE	137.27 ± 17.44^A^	146.87 ± 14.01^B^	153.51 ± 13.2^C^	<0.001		
	*P* value*	0.017	<0.001	<0.001	—		
Reading and understanding	VBE	36.74 ± 6.69^A^	40.09 ± 4.96^B^	42.3 ± 3.34^C^	<0.001	<0.001	*F* = 27.01 *p* = 0.56
	BBE	33.47 ± 6.62^A^	37.71 ± 6.13^B^	39.21 ± 5.47^C^	<0.001		
	*P* value*	<0.001	0.002	<0.001	—		
Access to information	VBE	47.39 ± 11.36^A^	51.75 ± 8.30^B^	53.41 ± 7.15^C^	<0.001	0.028	*F* = 4.91 *p* = 0.005
	BBE	46.95 ± 9.2^A^	48.18 ± 8.01^B^	50.52 ± 7.79^C^	<0.001		
	*P* value*	0.75	0.001	0.005	—		
Evaluation of information	VBE	23.42 ± 5.69^A^	26.42 ± 3.58^B^	29.03 ± 1.65^C^	<0.001	<0.001	*F* = 4.12 *p* = 0.02
	BBE	21.47 ± 4.9^A^	23.36 ± 3.99^B^	24.99 ± 3.64^C^	<0.001		
	*P* value*	0.007	<0.001	<0.001	—		
Decision making and application	VBE	36.74 ± 9.1^A^	39.93 ± 7.66^B^	44.05 ± 5.71^C^	<0.001	>0.001	*F* = 5.90 *p* = 0.003
	BBE	35.38 ± 7.44^A^	37.62 ± 7.03^B^	38.79 ± 5.35^C^	<0.001		
	*P* value*	0. 22	0. 021	<0.001	—		

* The results of the independent‐sample *t* test.

** The results of the repeated measures analysis of variance; A, B, and C: Significant pairwise difference between time points in each group determined through Bonferroni's method.

## 4 DISCUSSION

This study compared the effects of premarital BBE and VBE on couples’ RHL. Findings revealed that both BBE and VBE significantly improved RHL while VBE was significantly more effective than BBE. In agreement with our findings, a study showed that premarital education significantly improved couples’ RH‐related knowledge and attitude.^35^ A randomized clinical trial study from Japan also found Health literacy improved after a two‐week e‐learning plan.^36^ Studies reported that education using digital media significantly improved women's health‐related behaviors as well as Internet‐based method meaningfully enhanced adolescents’ knowledge ofSRH.^37^, ^38^ Moreover, a study showed that both webinar and group discussion methods significantly improved healthcare workers’ attitude and performance respecting fertility‐related counseling provision. That study highlighted that education through the webinar method can also improve critical thinking, decision‐making, and psychomotor skills.^39^ The more significant effects of VBE compared with BBE in the present study are attributable to the positive attributes of VBE, including easy accessibility, flexibility,^40^, ^41^, ^42^ low cost, possibility to protect learner privacy, learners’ greater opportunityto review educational materials,^43^ learners’ greater abilityto understand sensitive issues such as sexual issues that may be unpleasant to explain in face‐to‐face educational sessions, and the opportunity to respond learners’ questions through virtual methods. Moreover, virtual education has turned into a part of contemporary daily life and has significantly modified interpersonal interactions.^44^ Therefore, it can be used to effectively provide quality premarital education to prevent possible RH‐related problems and complications. Contrary to our findings, two studies reported the ineffectiveness of face‐to‐face premarital education on RH‐related knowledge and attitude.^31^, ^32^ This ineffectiveness might have been due to the limited opportunity for participants to ask their questions after educational sessions.^32^

Our findings also showed that the mean score of the reading and understanding dimension of RHL significantly increased in both BBE and VBE groups and there was no significant difference between the groups. This implies that both BBE and VBE can significantly improve RHL. In line with this finding, previous studies reported that educational interventions (such as group counseling, computer‐based education, and mobile‐based education) significantly improved HR‐related knowledge, understanding, and literacy.^30^, ^44^, ^45^ A study also highlighted that social networks facilitated access to health‐related information and hence, helped improve HL.^46^

We also found a significant increase in the mean score of the access to information dimension of RHL in both groups, though the amount of increase in the VBE group was significantly more than the BBE group. Similarly, a study showed that health education through social media significantly increased the mean scores of all four dimensions of RHL among female adolescents.^30^ Another study reported that technology‐based education significantly increased students’ HL andintroduced virtual media as the most accessible and usable source of information about sexuality and RH.^47^ The greater effectiveness of VBE in the present study compared with BBE may be due to the greater accessibility of educational materials in this method.

Study findings also showed that the mean score of the evaluation of information dimension significantly increased in both groups and the amount of increase in the VBE group was significantly more than the BBE group. Previous studies reported that better evaluation and understanding of web‐based health‐related information were associated with greater HL,^48^ more active use of different health‐related information and different information search strategies, and more precise evaluation of health‐related information.^49^ More effective use of virtual media by individuals with greater HL creates new educational needs and more clearly highlights the importance of virtual education for different age groups, particularly for young people in marriage age.

Our findings also indicated that although both BBE and VBE significantly increased the mean score of decision‐making and application dimension of RHL, the effect of VBE on this dimension was significantly more than BBE. In agreement with our findings, a study showed that mobile‐based education significantly increased sexual and reproductive HL among adolescents and significantly improved their information application, decision‐making ability, and ability to more appropriately use HL.^30^ HL improvement is associated with significant improvement in the ability to collect and interpret health‐related information, significant improvement in attitudes towards health‐promoting behaviors, greater preoccupation with these behaviors, and greater possibility of engagement in them.^50^

## 5 IMPLICATION

Due to the greater effectiveness of the virtual method, in improving RHL, this method can be used for preparing content of the premarital training sessions.

### 5.1 Study limitations

One of the limitations of this study was the lack of face‐to‐face interaction between instructors and learners. Moreover, participants were not obliged to use the provided education. We attempted to manage these limitations by encouraging participants to actively participate in the educational program, protecting their privacy, answering their questions, and providing them with the opportunity to receive private counseling. Another limitation of the study was the significant between‐group difference respecting the pretest means score of RHL.

## 6 CONCLUSION

This study concludes that both BBE and VBE are effective in significantly improving RHL among engaged couples, while the effects of VBE on the access to information, evaluation of information, and decision making and application dimensions of RHL are significantly more than BBE. Given the significant positive effects of premarital education on couples’ RH‐related knowledge, attitudes, and behaviors, the use of effective educational methods is essential to maximize its effectiveness.

The limitations related to the education of sensitive issues to adolescents and young people in the sociocultural context of Iran highlight the importance of using virtual methods to improve their RHL.

## AUTHOR CONTRIBUTIONS

SB and SO contributed to the concept and design of the study; SB collected the data, SK contributed to the data analysis; FM and HM supervised the data collection, SO and SK contributed to the interpretation of the data; SO drafted the manuscript and prepared the final version of the manuscript. All the authors met the criteria for authorship and were listed as co‐authors on the title page.

All authors have read and approved the final version of the manuscript. Corresponding author have full access to all of the data in this study and takes complete responsibility for the integrity of the data and the accuracy of the data analysis.

## CONFLICT OF INTEREST STATEMENT

The authors declare no conflicts of interest.

## ETHICS STATEMENT

The study design was approved by Ethics Committee (IR. MUBABOL. REC.1400.068). Written informed consent was taken from all the participants. All methods were carried out in accordance with relevant guidelines and regulations.

## CONSENT FOR PUBLICATION

Not applicable.

## TRANSPARENCY STATEMENT

The lead author Shabnam Omidvar affirms that this manuscript is an honest, accurate, and transparent account of the study being reported; that no important aspects of the study have been omitted; and that any discrepancies from the study as planned (and, if relevant, registered) have been explained.

## Supporting information

Video 1: Key points about having a stable and happy family.

Video 2: An overview of the genital system, sexual function, and contraception.

Video 3: RH.

Video 4: Critical pregnancy care and childbearing (Table 1).

Supporting information.

## Data Availability

The data that support the findings of this study are available from the corresponding author upon reasonable request. The datasets used and/or analyzed during the current study are available from the corresponding author upon reasonable request.
